# Adaptive radiation therapy strategies in the treatment of prostate cancer patients using hypofractionated VMAT

**DOI:** 10.1002/acm2.13415

**Published:** 2021-11-16

**Authors:** Pawel Siciarz, Boyd McCurdy, Nikesh Hanumanthappa, Eric Van Uytven

**Affiliations:** ^1^ Department of Medical Physics CancerCare Manitoba Winnipeg Manitoba Canada; ^2^ Department of Physics and Astronomy University of Manitoba Winnipeg Manitoba Canada; ^3^ Department of Radiology University of Manitoba Winnipeg Manitoba Canada

**Keywords:** adaptive radiation therapy, deformable image registration, hypofractionation, offline plan adaptation, online plan adaptation, prostate cancer, VMAT

## Abstract

**Purpose:**

To perform a comprehensive evaluation of eight adaptive radiation therapy strategies in the treatment of prostate cancer patients who underwent hypofractionated volumetric modulated arc therapy (VMAT) treatment.

**Material and methods:**

The retrospective study included 20 prostate cancer patients treated with 40 Gy total dose over five fractions (8 Gy/fraction) using VMAT. Daily cone beam computed tomography images were acquired before the delivery of every fraction and then, with the application of deformable image registration used for the estimation of daily dose, contouring and plan re‐optimization. Dosimetric benefits of the various ART strategies were quantified by the comparison of dose and dose‐volume metrics derived from treatment planning objectives for original treatment plan and adapted plans with the consideration of target volumes (PTV and CTV) as well as critical structures (bladder, rectum, left, and right femoral heads).

**Results:**

Percentage difference (ΔD) between planning objectives and delivered dose in the D_99% _> 4000cGy (CTV) metric was −3.9% for the non‐ART plan and 2.1% to 4.1% for ART plans. For D_99% _> 3800cGy and D_max _< 4280cGy (PTV), ΔD was −11.2% and −6.5% for the non‐ART plan as well as −3.9% to −1.6% and −0.2% to 1.8% for ART plans, respectively. For D_15%_ < 3200 cGy and D_20%_ < 2800 cGy (bladder), ΔD was −62.4% and −68.8% for the non‐ART plan as well as −60.0% to −57.4% and −67.0% to −64.0% for ART plans. For D_15%_ < 3200 cGy and D_20%_ < 2800 cGy (rectum), ΔD was −11.4% and −8.15% for non‐ART plan as well as −14.9% to −9.0% and −11.8% to −5.1% for ART plans.

**Conclusions:**

Daily on‐line adaptation approaches were the most advantageous, although strategies adapting every other fraction were also impactful while reducing relative workload as well. Offline treatment adaptations were shown to be less beneficial due to increased dose delivered to bladder and rectum compared toother ART strategies.

## 1 INTRODUCTION

Radiation therapy is a major treatment option for patients with prostate cancer. However, variations in the patients’ anatomy during radiotherapy treatment can present challenges. Numerous studies show that the variable location of the prostate, bladder filling, and pockets of gas often present in the rectum might significantly compromise dose coverage of the target structure and increase the dose delivered to critical organs.^1^, ^2^, ^3^, ^4^, ^5^

Hypo‐fractionated radiotherapy, delivering fewer, higher fraction doses increases the dosimetric impact of anatomic variability compared to conventional fractionation schemes. For prostate cancer patients, moderate hypofractionated (70 Gy in 28 fractions, 2.5 Gy/fraction) intensity modulated radiation therapy (IMRT) and volumetric modulated arc therapy (VMAT) were proven to keep early normal tissue toxicity at acceptable levels.^6^, ^7^ More aggressive hypofractionation delivering 33.5–37.5 Gy in five fractions has also been shown to achieve acceptable toxicity and quality of life,^9^, ^10^ even for high risk and very high risk (including node‐positive) prostate cancer patients.

The safe and effective delivery of high radiation doses in hypofractionated schemes requires a high level of precision, but inter‐ and intra‐fractional patient anatomical variation is present and known to compromise dosimetric aspects of the treatment.^8^, ^9^ Adaptive radiation therapy (ART) strategies, in particular on‐line ART, have the ability to account for systematic anatomic changes of prostate swelling as well as random anatomic changes such as inter‐ and intra‐fraction bladder and rectal filling, in addition to independent movement and deformation of multiple targets.^8^, ^10^ The necessity and the benefits of ART application in stereotactic body radiation therapy (SBRT) prostate treatments have been shown in other recent studies.^9^, ^10^, ^11^, ^12^ It should also be noted that existing image guided radiation therapy (IGRT) techniques, although allowing for prostate motion management, have some limitations. For example, they might require an invasive procedure carrying the risk of bleeding, infection, and discomfort for the patient (radiopaque intraprostatic fiducial markers method). Another prostate IGRT example is a technique that utilizes inserted electromagnetic transponders. In this case, patient eligibility criteria are very strict as only patients without hip prosthesis, metal implants, peacemaker, or other electromagnetic devices are eligible, as well as relatively thinner patients due to the maximum range of the beacon detection by a required external array (reading) device.^13^

Adaptive radiation therapy is currently an active area of research, and there are still many novel ART approaches that have not been explored yet but could make a significant contribution to the field. The current study focuses on a comprehensive evaluation of several ART methods that have not been explored for the prostate VMAT hypofractionation schemes examined here. The purpose of this research was to retrospectively investigate eight adaptive radiation therapy strategies (including both online and offline scenarios) for hypofractionated VMAT treatments based on imaging and treatment plan data of 20 prostate cancer patients with the application of deformable image registration (DIR). The online and offline adaptations considered were compared to the *non‐ART* (not adapted) delivery scenario.

## 2 MATERIALS AND METHODS

### 2.1 Patient data

The imaging and treatment planning data for twenty prostate cancer patients, with an average age of 77 (±7) years were retrospectively used for this study. The study was approved by the local research ethics board (University of Manitoba).

All 20 patients had previously received a 40 Gy/5 fraction treatment regimen and were treated at CancerCare Manitoba. One pre‐treatment, planning computed tomography (pCT) imaging scan, and five sets of on‐treatment cone beam CT (CBCT) imaging scans were obtained for each patient. CBCT images were acquired during each treatment fraction right before radiation delivery to ensure proper patient positioning. Anatomic structures considered in the dosimetric analysis included the clinical target volume (CTV), the planning target volume (PTV = CTV+0.5 cm margin) as well as organs‐at‐risk (OARs) ‐ bladder, rectum, and femoral heads. An experienced radiation oncologist segmented these structures on both CT and CBCT imaging data sets. These structures were also used for plan adaptation and optimization purposes.

The pCT images, at 512 × 512 pixels, were obtained with a spatial resolution of 1.17 mm × 1.17 mm per pixel and 3.0 mm slice thickness (total of ∼210 slices) on a Philips Brilliance Big Bore CT scanner. The CBCT images, at 384 × 384 pixels, were obtained with a spatial resolution of 1.17 mm × 1.17 mm per pixel and 2.5 mm slice thickness (total of 64 slices) using an OBI Cone‐Beam CT unit (Varian Medical Systems, Palo Alto, CA).

### 2.2 Dose delivery and treatment planning

The treatment was delivered using VMAT with two full arcs. Every patient was treated with a full bladder and empty rectum as per local clinical protocol. The intent of the radiation therapy was curative for all patients. Treatment plans were created in the external beam planning module of the eclipse treatment planning system, version 13.6 (Varian Medical System, Palo Alto, CA, USA). Dose and dose‐volume objectives for the radiation treatment are summarized in Table 1. The beam energy and the maximum dose rate for both arcs were 6MV and 1000MU/min (SRS mode). All the *non‐ART* plans were normalized to the dose received by 95% of the PTV volume. Specifically, the dose was determined based on the 95% of the PTV volume on the dose volume histogram of the original treatment plan. The *non‐ART* plans were not normalized.

**TABLE 1:** Treatment planning objectives for target and organs‐at‐risk (OARs) structures

Structure	Prescription			Fraction dose (cGy)	Total dose (cGy)	Planning objective symbols*
CTV	At least	99.0	% receives more than	800.0	4000.0	D_99% _> 4000 cGy
PTV	At least	99.0	% receives more than	760.0	3800.0	D_99% _> 3800 cGy
PTV	Maximum dose		is	856.0	4280.0	D_max _< 4280 cGy
Rectum	At most	15.0	% receives more than	640.0	3200.0	D_15%_ < 3200 cGy
Rectum	At most	20.0	% receives more than	560.0	2800.0	D_20%_ < 2800 cGy
Bladder	At most	15.0	% receives more than	640.0	3200.0	D_15%_ < 3200 cGy
Bladder	At most	20.0	% receives more than	560.0	2800.0	D_20%_ < 2800 cGy
Femur‐RT	At most	5.0	% receives more than	560.0	2800.0	N/A
Femur‐LT	At most	5.0	% receives more than	560.0	2800.0	N/A

*The percentage deviations from these objectives for all adaptive radiation therapy (ART), *planned* and *non‐ART* plans have been illustrated in Figure 6b for CTV and PTV as well as in Figure 7b for Bladder and Rectum. Femoral heads (right ‐ RT; left ‐ LT) were not included in the plan evaluation as the radiation doses for all plans were significantly below the planning objective thresholds.

Abbreviations: CTV, clinical target volume; PTV, planning target volume.

### 2.3 Adaptive radiation therapy

#### 2.3.1 DIR

Daily CBCT images were acquired before the delivery of every fraction. Planning CT images were then registered to CBCT data sets using a Bspline‐based^14^ automated DIR algorithm available in Velocity AI, version 3.2 (Varian Medical System, Palo Alto, CA, USA). Deformed pCT images (‘dCT’) were then used for daily dose estimation with respect to the treatment plan (adapted and non‐adapted) that was delivered to the patient during a particular fraction. Since our study was retrospective, the plan delivery was simulated (i.e., it was not actually delivered to the patient). Contours that allowed for the estimation of the dose delivered to the considered anatomical structures were delineated by an experienced physician on CBCT and then propagated to daily dCT image scans. To adapt to the current patient anatomy, and as needed for some of the strategies studied here including adaption while accounting for the dose delivered in the previous fraction(s), the subsequent fraction treatment plans were re‐optimized simulating offline or/and online scenarios.

#### 2.3.2 Plan optimization

For the optimization of VMAT plans in this study, the progressive resolution optimizer,^15^ version 10.0.28 was used, while for dose calculation, the AAA algorithm (v.10.0.28) was utilized with a 0.25 cm calculation grid resolution. The total dose delivered to the patient after performing a given plan adaptation was estimated by mapping daily doses back to the reference (planning CT) image using an inverted deformation vector field obtained through DIR and then by performing dose accumulation using the Velocity AI software. The objectives were consistent and unmodified throughout the optimization process relative to the *Planned* plans.

#### 2.3.3 Adaptive radiation therapy strategies

For this study, online, offline, and dose feedback (DF) approaches were examined by simulation using the available daily anatomical CBCT data set. Online plan adaptations were simulated to occur immediately before a dose delivery while offline modifications were simulated to occur between fractions n and n+1. DF adaptation was simulated as an offline strategy and utilized the dose delivered in the previous fraction to guide a plan adaptation (re‐optimization) for the next fraction. Overall, eight adaptive radiation therapy strategies were simulated for all patients as described below.

1. *DF 2–4* – A combination of the *non‐ART* treatment plan and DF adaptation. In the 1st, 3rd, and 5th fraction, the *non‐ART* plan was delivered. In the 2nd and 4th fraction, the *non‐ART* plan was re‐optimized based on the dose delivered during the previous fraction. The reasoning behind performing re‐optimization only during the 2nd and 4th fraction is as follows: The second fraction is the first fraction that can use the feedback from the dose delivered in the previous (1st) fraction. During the third fraction, the *non‐ART* treatment plan was delivered because the dose delivered during the 2nd fraction accounted for dose discrepancies resulting from dose delivery during the first fraction, and thus up to the 3rd fraction the plan was assumed to be delivered optimally. Therefore, the time‐consuming DF adaptation was not used during the 3rd fraction. To examine the performance of DF applied to every fraction (for example with the availability of in vivo patient dosimetry), we have also tested a continuous DF adaptation (*Cont.+DF* approach).
  The dose delivered in the previous fraction was incorporated in the optimization process for the current fraction using the “dose‐based” plan optimization module of eclipse. Before the start of the optimization process, the dose delivered in the previous fraction was mapped to the patient's anatomy of the current fraction using DIR. Once the optimization was initiated, the optimizer compensated for regions of lower than or higher than intended dose by delivering a higher/lower dose to those regions, so that the total accumulated dose delivered during the previous and the current fraction would meet the treatment plan objectives. The application of the DF in the subsequent plan adaptation scenarios was performed in the same manner.
2. *Offline* – based on the offline adaptation of the *non‐ART* treatment plan. In the 1st fraction, the *non‐ART* plan was delivered. To deliver the dose during the 2nd, 3rd, 4th, and 5th fraction, the treatment plan was adapted by plan re‐optimization using the dCT from the previous fraction (obtained based on the previous fraction's CBCT). As it implies, in this adaptation scenario, only changes in the patients’ anatomy detected on the daily CBCT image data set relative to the planning CT images were accounted for. The dose delivered during the previous fraction was not considered as described in approach (i).
3. *Offline + DF* – based on the combination of offline adaptation of the *non‐ART* plan and DF adaptation. In the 1st fraction, the *non‐ART* plan was delivered. In the 2nd fraction, the treatment plan was re‐optimized using the daily image data sets (daily dCT) and the dose delivered during the previous fraction (DF adaptation). In the 3rd fraction, the dose was delivered using an offline adapted plan (based on the previous fraction image data sets). In the 4th and 5th fractions, DF and offline adaptation were applied, respectively.
4. *Online* – based on the daily online adaptation of the *non‐ART* plan. In all fractions, before the daily dose was delivered, the treatment plan was re‐optimized according to the patient's anatomy just before treatment delivery.
5. *Online + DF* – based on the combination of daily online adaptation and DF adaptation. In the 1st, 3rd, and 5th fraction, online adaptation was performed, while in the 2nd and 4th, fraction DF adaptation was performed.
6. *Cont. + DF* – based on the combination of continuous DF adaptation. In the 1st fraction, the *non‐ART* plan was delivered. During the remaining, 2nd–5th fractions, the treatment plan was adapted using the patient's daily anatomy. The plan re‐optimization was performed based on the total dose delivered over all the previous fractions.
7. *Online 1‐3‐5* – based on the daily online adaptations and *non‐ART* treatment plan. In the 1st, 3rd, and 5th fraction, the treatment plan was adapted using an online approach, while in the 2nd, and 4th fraction, the *non‐ART* treatment plan was delivered.
8. *Offline+Online* – based on the combination of online and offline adaptations. In the 1st, 3rd, and 5th fraction, the treatment plan was adapted using an online approach while in the 2nd, and 4th fraction using offline plan adaptation.

The purpose behind creating various adaptive radiation therapy strategies was to find an optimal solution for treatment plan adaptation which minimizes the negative impact of changes in the patient's anatomy on the accuracy of the delivered dose, while also minimizing the time it would take to perform such adaptations in a clinical environment. For example, the rationale behind examining the *Online 1‐3‐5* strategy, where online adjustments of the treatment plan were performed every second fraction instead of every fraction, was to decrease the total time of adaptation relative to a full *Online* strategy, where online adjustments of the treatment plan were performed every fraction. Another example is a *DF 2–4* strategy (where *DF *= DF). Incorporating a DF step allows accounting for potentially inaccurate dose delivery in the previous fraction and the change in the patient's anatomy because optimization involving the DF step is performed on the daily imaging data. Although the implementation of those steps will increase total treatment time, it is expected that the improved accuracy in the dose delivery will justify the additional workload.

In this study, we define the reference *non‐ART* plan as the one that was created based on the pCT data and was delivered at every treatment fraction without modifications (dose delivered was calculated based on the CBCT and mapped back to the reference pCT image). This is an estimate of what dose is actually delivered by the conventional *non‐ART* approach.

*Planned* delivery reflects the intended (ie. prescribed) dosimetry of the treatment plan, as approved by the radiation oncologist.

### 2.4 Evaluation of adaptive radiation therapy strategies

The dosimetric effectiveness of adaptive radiation therapy strategies was evaluated using a variety of dose and dose‐volume metrics for target and OARs as specified in Table 2. Metrics were selected based on the relevant literature to ensure general applicability.^16^, ^17^, ^18^, ^19^, ^20^, ^21^, ^22^, ^23^ The values of each metric were associated with anatomic structures, ART strategies, and the reference plan. Where applicable, the evaluation metrics for ART plans were presented relative to the reference plan as well to quantify the dosimetric benefit of applying plan adaptations compared to the situation where the adaptations were not incorporated in the treatment process.

**TABLE 2:** Quantitative metrics used for evaluation of adaptive radiation therapy strategies. Apart from all listed metrics, maximum dose (D_max_), mean dose (D_mean_), and minimum dose (D_min_) for each structure were determined as well

	Bladder &	Femur‐LT &
CTV & PTV	Rectum	Femur‐RT
D_1%_ (cGy)	D_1%_ (cGy)	D_1%_ (cGy)
D_2%_ (cGy)	D_1cc_ (cGy)	D_1cc_ (cGy)
D_5%_ (cGy)	D_2%_ (cGy)	D_2%_ (cGy)
D_50%_ (cGy)	V_15%(6_ _Gy)_	D_5%_ (cGy)
D_95%_ (cGy)	V_20%(8_ _Gy)_	
D_98%_ (cGy)	V_50%(20_ _Gy)_	
D_99%_ (cGy)	V_80% (32_ _Gy)_	
V_100%_	V_95% (32_ _Gy)_	
V_105%_		
HI* (%)		
CI** (%) (PTV only)		
V_95%_ (PTV only)		

D_v%_ ‐ minimum dose delivered to the “hottest” v% of the volume, D1cc – minimum absolute dose for the “hottest” 1cm^3^ of the volume, V_d%_ ‐ a volume that received d% or more of the prescription dose expressed as a percentage of volume, HI – homogeneity index calculated as a ratio (D_2%_ ‐ D_98%_)/D_50%_,^24^ CI – conformity index calculated as a ratio V_95%_/Volume of PTV.^25^

Abbreviations: CTV, clinical target volume; PTV, planning target volume.

The calculation of the percentage of plans that passed the treatment planning criteria for CTV, PTV, and OAR structures was reported as well. Due to their clinical relevance, the percentage deviations from the treatment planning objectives were also reported. Importantly, our conclusions with respect to superiority and inferiority of particular ART strategies relative to other dose delivery approaches were mostly driven by analysis of treatment planning objectives. Specifically, the larger the passing rate, the more clinically feasible we considered a given ART approach. For CTV and PTV, the smaller the absolute percentage deviations from the planning objectives were considered better. For OARs, the smaller percentage deviations were considered better. The large number of other dose‐volume metrics (Table 2) that we have included in our study provide a more comprehensive view on the dosimetry of all considered plans but were not considered in the exact treatment planning objectives used to derive the plans (these are included in Table 1).

The statistical significance of the results comparing the *non‐ART* plan to all the other plans was determined using paired *t*‐tests, using the *p*‐value associated with a 95% confidence level. The time efficiency of the best performing ART method was also reported. The time required for each ART strategy was estimated with the consideration of plan optimization, dose calculation, and DIR procedures.

### 2.5 Qualitative assessment of image registration

In order to ensure that the image registration did not introduce any major errors in terms of the patient's anatomy deformations, a qualitative (i.e., visual) evaluation of deformed images and deformable vector fields was performed. In particular, the deformed images were compared to the target images by using image overlays, checkboard filters, dynamic magnifying window focusing on soft tissue and bone tissue alignment as well as external body contour, for every registration. The analysis of the vector fields included the inspection of the deformed grid that reflected the magnitudes and directions in the field. The inspection was conducted using tools available in the commercial image registration software Velocity AI (as specified in the Section 2.3.1).

## 3 RESULTS

### 3.1 Maximum, minimum, and mean doses

**FIGURE 1:**
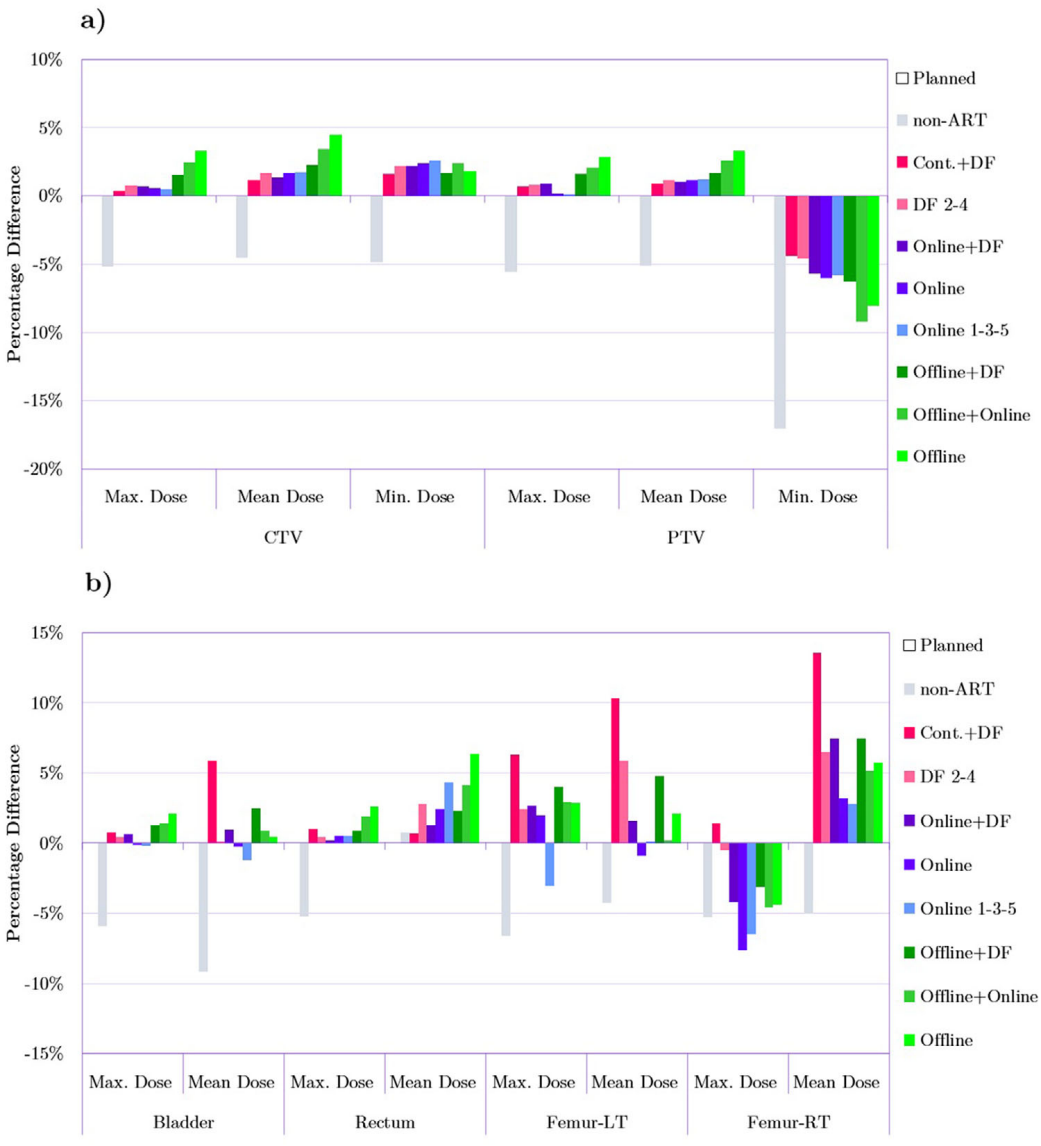
Comparison of D_max_ and D_mean_, and D_min_ metrics between the *Planned* plan as well as non‐adaptive radiation therapy (ART) and adapted plans for (a) target structures and (b) for OARs. Bars represent percentage differences (averaged over 20 patients) in particular metrics relative to the *Planned* treatment. The measure of 0% on the y‐axis is the reference point reflecting the value of a given metric for the *Planned* plan (i.e., reference plan).

Figure 1 shows the percentage differences relative to the *non‐ART* (i.e., reference, not adapted) plan in D_max_, D_mean,_ and D_min_ for CTV, PTV (Figure 1a) as well as in D_max_ and D_mean_ for OARs (Figure 1b). D_min_ for OARs was calculated as well but due to their limited applicability were not included in the results. Appendix (Table A1) contains the detailed tabular data for Figure 1 including D_min_ for OAR and standard deviations for all metrics.

All the metrics in Figure 1a indicate that values of D_max_, D_mean_, and D_min_ in the case of CTV and PTV for adapted plans were closer to the original (*P* *lanned*) plans compared to the unadapted reference plan. Overall, in terms of dose metrics reported in Figure 1a, continuous DF adaptation outperformed other ART strategies and had a performance close to online adaptations. The *Online* and *Online 1‐3‐5* plans scored as well as *Planned* plans in terms of the maximum dose delivered to PTV. The most apparent difference between adapted plans and the dose delivered by the non‐adapted plan was reflected in the value of the minimum dose to PTV. In particular, the unmodified plan resulted in the delivery of approximately 17% lower D_min_ to PTV compared to the planned minimum dose. Most adapted plans significantly improved the delivered dose in terms of this evaluation metric. Overall, it can be noticed that *non‐ART* plans delivered a lower radiation dose to both CTV and PTV in terms of D_max_, D_mean_, and D_min_.

Figure 1b for the OARs shows that D_max_ for both bladder and rectum in the *non‐ART* plan differed from the planned dose by around 5% (decrease). However, adapted plans were able to closely match the *Planned* plans decreasing the difference in D_max_ to around 1%–2%. In contrast, the mean dose for bladder and rectum showed larger variations for the adapted plans relative to the *non‐ART* plan. In the case of the bladder, the majority of adaptations increased the D_mean_ by less than 5%. Only *Cont.+DF* plans escalated the mean dose by around 6%. *Online* and *Online 1‐3‐5* plans were able to slightly reduce the D_mean_ for the bladder relative to the planned dose. The rectum received approximately 5% lower mean dose upon delivery of the *non‐ART* plan compared to the planned dose. As can be seen, *Cont.+DF*, *Offline+DF*, and online strategies were able to decrease that difference to roughly 0.5%–2%. The dose to the femoral heads was spared the most through the application of *Online* and *Online 1‐3‐5*. As for the right femoral head, those two online adaptation techniques delivered maximum doses significantly smaller even compared to the planned dose. However, the *Cont.+DF* adaptation resulted in higher than intended dose to both left and right femoral heads – a 6% and over 10% increase in the mean dose, respectively.

### 3.2 Dose‐volume metrics

#### 3.2.1 Target structures

**FIGURE 2:**
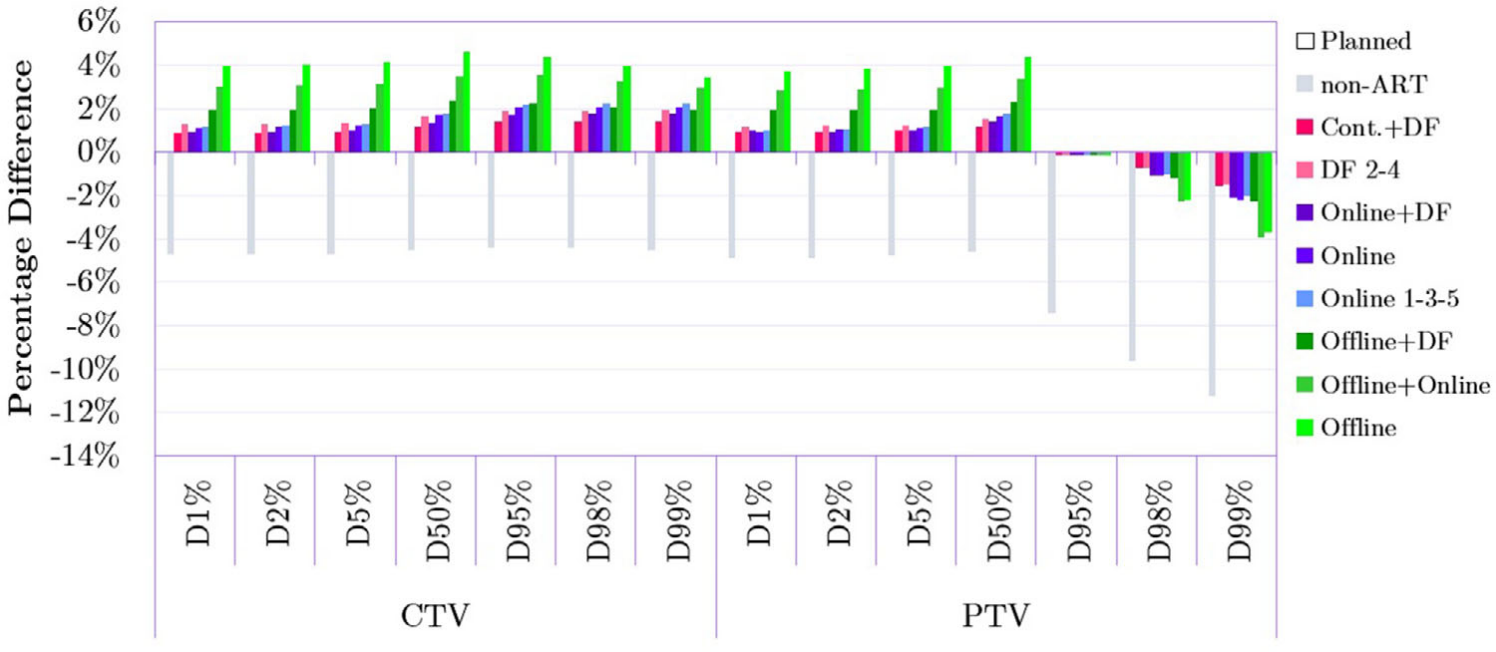
Comparison of dose‐volume metrics between the *Planned* plan as well as non‐adaptive radiation therapy (ART) and adapted plans for the clinical target volume (CTV) and planning target volume (PTV). Bars represent percentage differences (averaged over 20 patients) in particular metrics relative to the *Planned* treatment. The measure of 0% on the y‐axis is the reference point reflecting the value of a given metric for the *Planned* plan.

Figure 2 shows the relative values of dose‐volume metrics that were calculated within the evaluation of adaptive radiation therapy strategies for the CTV and PTV. Overall, the dose delivered with the various adaptive strategies was consistently closer to the planned dose compared to the *non‐ART* approach. In that regard, *Offline*+*Online* and *Offline* plans demonstrated the lowest while *Cont.+DF* and online adaptations demonstrated the highest dosimetric performance for both target structures. The PTV benefited from ART more than CTV as shown by D_95%_, D_98%_, and D_99%_, metric. Notably the values of these three metrics for *non‐ART* plans were approximately 4% lower for CTV and as much as 11% lower for PTV compared to *Planned* plans. Tabular data for Figure 2 with standard deviations are included in the Appendix (Table A2).

**FIGURE 3:**
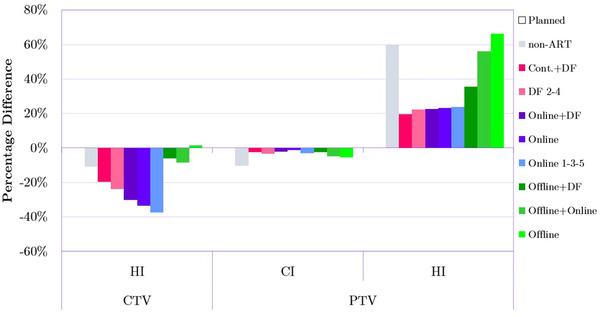
Comparison of homogeneity index (HI) and conformity index (CI) between the *Planned* plan as well as non‐adaptive radiation therapy (ART) and all adapted plans for clinical target volume (CTV) and planning target volume (PTV). Bars represent percentage differences (averaged over 20 patients) in the particular metrics relative to the *Planned* treatment. The measure of 0% on the y‐axis is the reference point reflecting the value of a given metric for *the Planned* plan.

When it comes to homogeneity index (HI) for CTV (Figure 3), the majority of adapted plans improved the homogeneity (i.e., a lower HI indicates a higher homogeneity level) of the dose distribution relative not only to the *non‐ART* plan but also to the *Planned* standard treatment. *Online* and *Online 1‐3‐5* delivered the highest benefit. For PTV, HI was also improved among all adapted plans. Specifically, online and DF plans performed similarly and outperformed offline strategies by around 20%–40%. Compared to the *non‐ART* dose delivery, CI for the planning target volume was approximately 10% higher when adapted plans were utilized. *Online* ART, in particular, very closely matched the *Planned* treatment. According to most metrics presented in Figure 3, *Offline* and *Offline+Online* adaptations were not meaningfully beneficial to the radiation treatment dosimetry.

#### 3.2.2 Organs at risk

**FIGURE 4:**
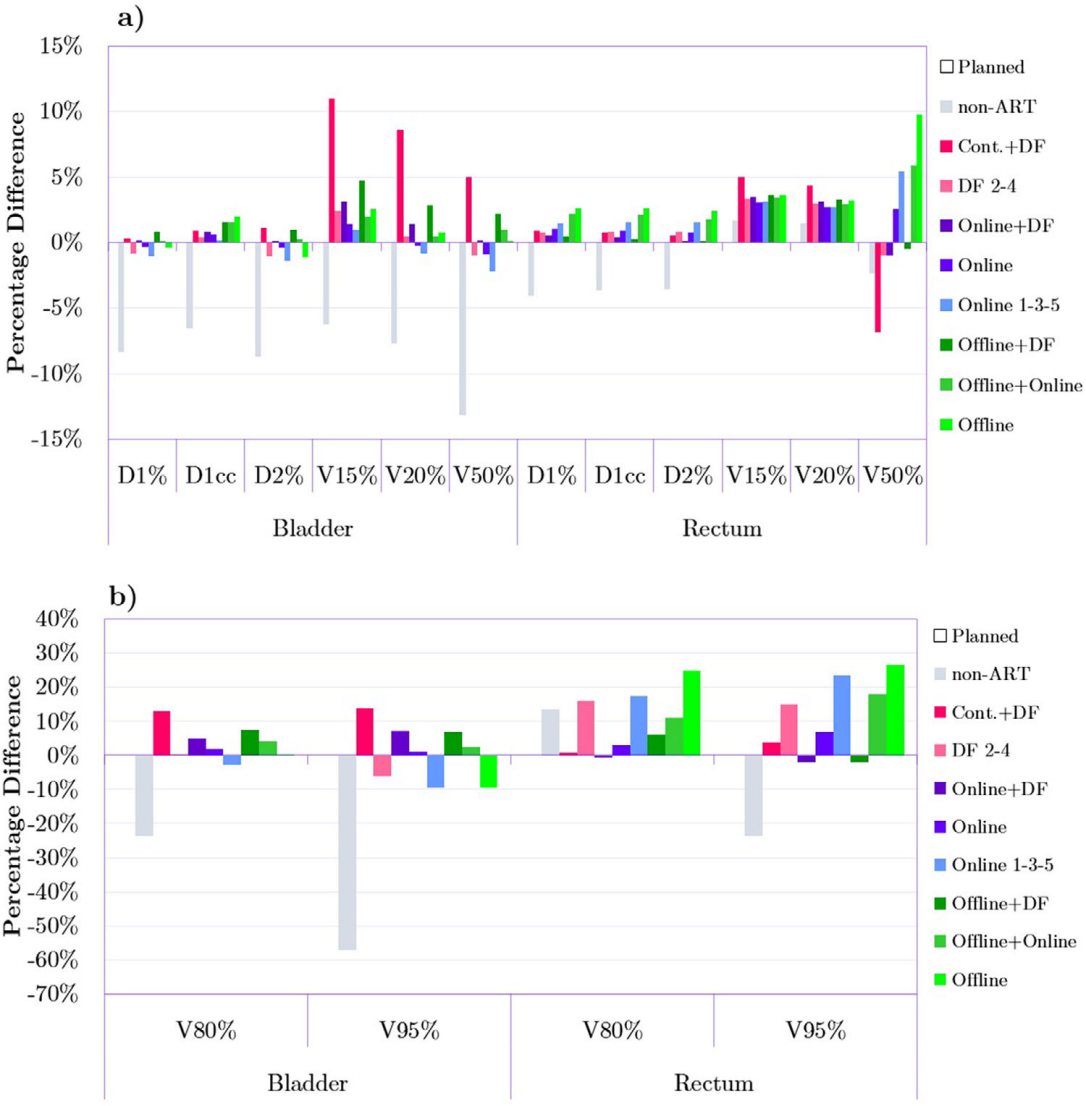
Comparison of dose‐volume metrics between the *Planned* plan as well as non‐adaptive radiation therapy (ART) and all adapted plans for bladder and rectum. Bars represent percentage differences (averaged over 20 patients) in particular metrics relative to the *Planned* treatment. The measure of 0% on the y‐axis is the reference point reflecting the value of a given metric for the *Planned* plan. The charts (a) and (b) were separated for better visualization of the results due to the large differences in y‐axis values between V_80%_, V_95%,_ and the rest of the metrics.

Figure 4 illustrates the dose‐volume metrics for the bladder and rectum. In the case of the bladder, D_1%_, D_1cc_, and D_2%_ did not differ significantly from the planned dose for adapted plans but were lower by approximately 7% for *non‐ART* plans. Only *Cont.+DF*, and a few *Offline* adaptations, showed slightly higher values compared to the *Planned* delivery. Larger variations in magnitude were observed in V_15%_, V_20%_, and V_50%_ metrics. The most desirable results were obtained through *Online* and *Online 1‐3‐5* strategies. *Cont.+DF* adaptive plans showed poor performance with large volumes receiving 15%, 20%, and 50% of the prescription dose as can be seen in Figure 4a. The same observation can be made in Figure 4b showing V_80%_ and V_95%_ metrics for the bladder. Considering results for the rectum, most ART strategies were able to closely match *Planned* values of D_1%_, D_1cc_, and D_2%_ (Figure 4a) as well as values of V_80%_ and V_95%_ (*Cont.+DF, Online+DF, Online and Offline+DF* in Figure 4b). For V_15%_ and V_20%_, the majority of adapted plans delivered nearly the same results except for *Cont.+DF* for which approximately 2% volume increase was noted for 15% and 20% dose prescription levels. Compared to adapted, the *non‐ART* plans were closer to the *Planned* plans for those two volume metrics. V_50%_ for *Planned* treatment was approximately the same as for *DF 2–4* and *Online+DF* adaptations. The *Cont.+DF* approach resulted in V_50%_ being around 7% lower compared to the *Planned* delivery.

**FIGURE 5:**
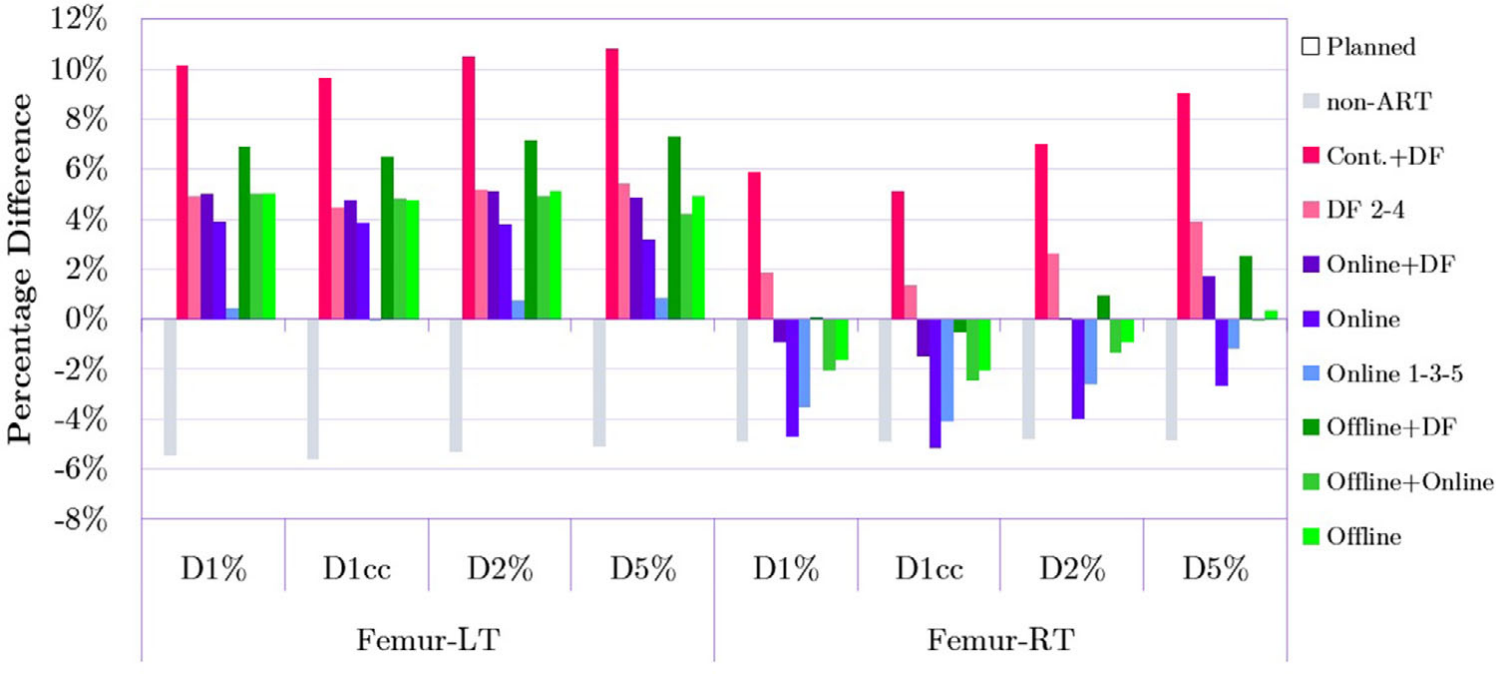
Comparison of dose‐volume metrics between the *Planned* plan as well as non‐adaptive radiation therapy (ART) and all adapted plans for left and right femoral heads. Bars represent percentage differences (averaged over 20 patients) in particular metrics relative to the *Planned* treatment. The measure of 0% on the y‐axis is the reference point reflecting the value of a given metric for the *Planned* plan. The lack of expected symmetry in the dose delivered by adapted plans to both femoral heads is explained in the Discussion section.

Figure 5 demonstrates that for the left femoral head only the *Online 1‐3‐5* plans were able to keep the D_1%_, D_1cc_, D_2%,_ and D_5%_ at the level close to the *Planned* treatment. All the other plan modifications, except for *non‐ART* resulted in doses higher than the *Planned* plans by around 4%–11%. In contrast, the majority of adapted plans (except for *Cont.+DF* and *DF 2–4*), especially *Online* and *Online 1‐3‐5*, deliver a lower D_1%_, D_1cc_, D_2%,_ and D_5%_ by up to 5% to the right femoral head compared to *Planned* dose delivery.

### 3.3 Comparison to treatment planning criteria

#### 3.3.1 Target structures

**FIGURE 6:**
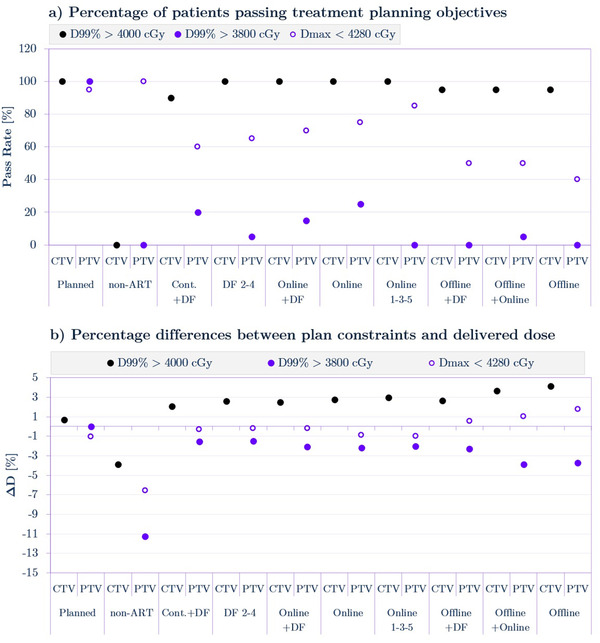
(a) Passing rates for clinical target volume (CTV) and planning target volume (PTV) for three treatment planning objectives. (b) Dose differences between the planning objectives and the dose delivered by the specific treatment plan.

Figure 6a demonstrates the percentage of patients for whom a given treatment plan met the treatment planning objectives specified in Table 1 (Section 2.2) for CTV and PTV structures. For CTV, all plans had at least a 90% passing rate except for *non‐ART* plans for which no patients passed CTV or PTV planning objectives. One hundred percent of patients passed the CTV criteria in the case of *DF 4‐2* and three *Online* adaptations. For the PTV, these same four strategies were able to achieve a 60%–80% passing rate for the D_max_ objective, while other ART approaches received 50% and lower rates. The D_99%_ criterion for PTV was very hard to reach even for well‐performing *Online* adaptations. The maximum passing rate was achieved by *Online* ART and was equal to slightly above 20%.

Figure 6b details the dose difference, ΔD, between the considered criteria and the value achieved by the plan. For the CTV, nearly all the plans were able to meet the treatment planning objective, and ΔD is positive ranging from 1%–4%. The dose difference in D_max_ for the PTV was approximately 1% for investigated ART approaches. It is noted that even though the passing rate for offline adaptations was lower in comparison to the rest of the ART strategies, the ΔD is, on average, positive for *Offline+DF*, *Offline+Online*, and *Offline* plans. This clearly shows that several patients delivered higher doses to the CTV so that it was able to cause the increase in the dose averaged over all 20 patients. The ΔD for D_99%_ objective for PTV ranged from around −4% in the case of *Offline* plans to approximately −2% for *Online* adaptations. Compared to all the ART approaches the *non‐ART* plans differs significantly from the *Planned* plans by −4% to −11% depending on the planning criteria.

In summary, the implementation of the majority of the ART strategies improved the overall passing rate and ΔD for most of the plans, especially for daily online adaptations compared to the delivery of an unchanged *non‐ART* treatment plans.

#### 3.3.2 Organs at risk

**FIGURE 7:**
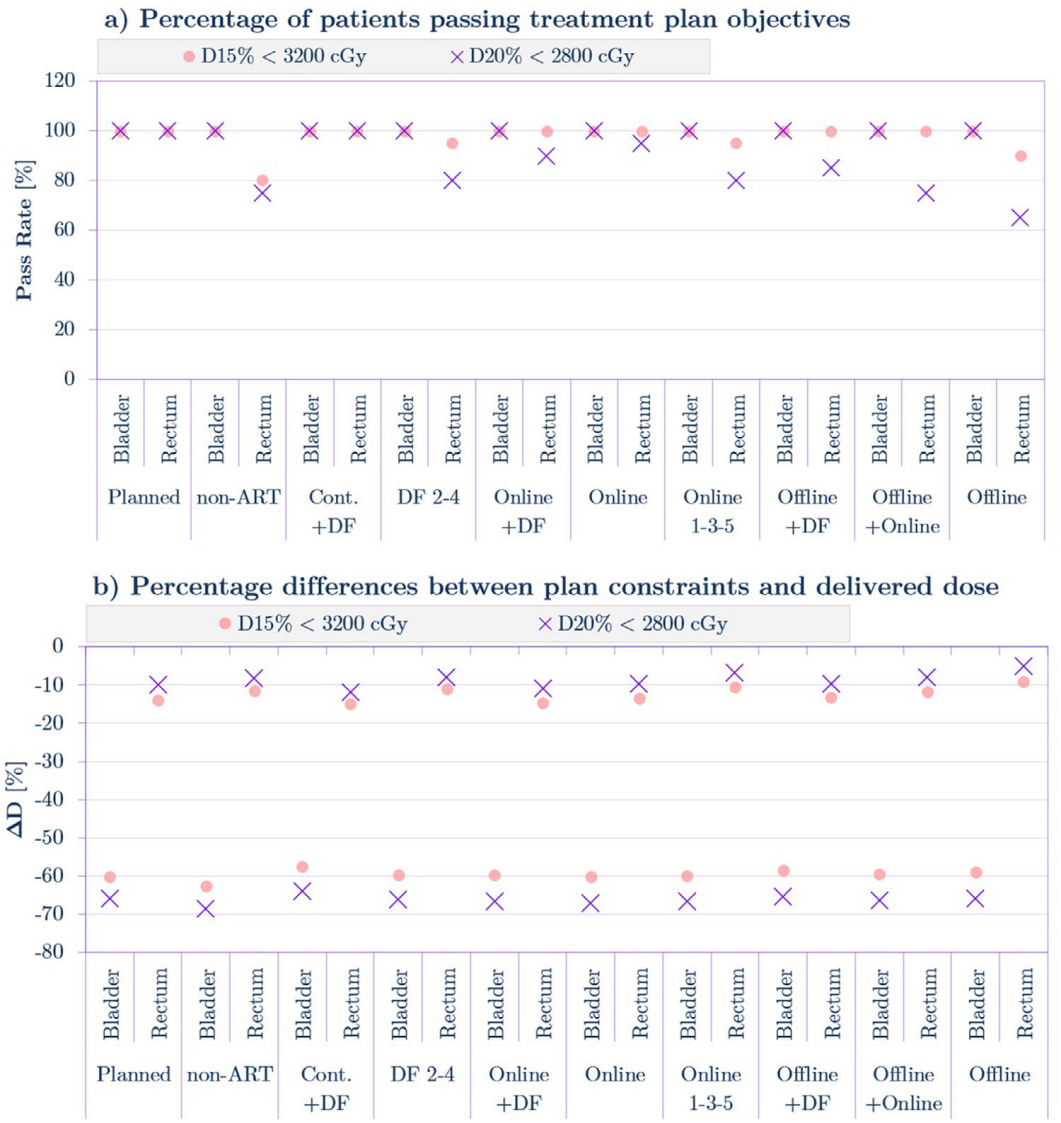
(a) Passing rates for organs at risk (OARs) in relation to the treatment planning objectives. (b) Dose differences between the planning objective and the dose value delivered by the specific treatment plan.

The passing rate presented in Figure 7a is equal to 100% across all the plans for the bladder in the case of both plan objectives. Consistently, Figure 7b shows that ΔD for the bladder is significantly (around 60%) below tolerance doses (D_15%_ and D_20%_). In the case of the rectum, the passing rate for three *Offline* adaptations and the *non‐ART* plan was in the range of 60%–85% for the D_20%_ metric and from 80% to 100% for the D_15%_ metric. For *Online* plans, the analogous range was from 80% to 95% for D_20%_ and from 95% to 100% for D_15%_. ΔD was negative for all the plans and had nearly the same magnitude (of 10%) for most of the adaptations for the D_15%_ and D_20%_ criteria. The absence of femoral heads in this analysis is addressed in the discussion section.

### 3.4 Statistical significance

As mentioned in the Materials and Methods section, the statistical significance of the results was calculated using paired, two‐tailed *t*‐tests. The analysis was based on the comparison of dose and dose‐volume metrics for target and OARs structures between the *non‐ART* plan and the other treatment strategies for all 20 patients. The relevant *p*‐values with a 95% confidence level are presented in Table 3 for D_max_, D_min_, and D_mean_ as well as in Appendix (Table A3) for the remaining metrics. Both tables also summarize the total number of metrics for which the test determined the statistical significance at the level of *p *< 0.05.

**TABLE 3:** The results of paired, two‐tailed *t*‐test for D_max_, D_min_, and D_mean._ The fields for which 0.01 < p ≤ 0.05 are highlighted in green (i.e., significant), while those with *p* ≤ 0.01 were highlighted in red (i.e., strongly significant)

			Cont.		Online		Online	Offline	Offline	
Structure	Metric	non‐ART	+DF	DF 2–4	+DF	Online	1‐3‐5	+DF	+Online	Offline
**CTV**	Maximum dose (%)	0.0000	0.4591	0.1416	0.2500	0.3321	0.2263	0.0089	0.0245	0.0007
	Mean dose (%)	0.0000	0.0136	0.0017	0.0058	0.0050	0.0002	0.0002	0.0022	0.0000
	Minimum dose (%)	0.0000	0.0059	0.0000	0.0001	0.0000	0.0000	0.0046	0.0000	0.0004
**PTV**	Maximum dose (%)	0.0000	0.1893	0.0796	0.1263	0.7760	0.9321	0.0037	0.0477	0.0026
	Mean dose (%)	0.0000	0.0132	0.0019	0.0060	0.0090	0.0004	0.0002	0.0036	0.0000
	Minimum dose (%)	0.0000	0.0223	0.0013	0.0234	0.0293	0.0013	0.0027	0.0018	0.0001
**Bladder**	Maximum dose (%)	0.0000	0.1813	0.3109	0.3115	0.9035	0.6836	0.0410	0.1550	0.0115
	Mean dose (%)	0.0007	0.1261	0.9888	0.7669	0.9129	0.4376	0.3785	0.7188	0.8258
	Minimum dose (%)	0.0000	0.0021	0.0002	0.0004	0.0002	0.0000	0.0003	0.0001	0.0001
**Rectum**	Maximum dose (%)	0.0000	0.0792	0.2371	0.7090	0.3807	0.1985	0.0515	0.0676	0.0046
	Mean dose (%)	0.5983	0.3821	0.0064	0.1046	0.0079	0.0003	0.0144	0.0063	0.0000
	Minimum dose (%)	0.1078	0.5133	0.4591	0.4791	0.4282	0.4094	0.5968	0.5261	0.6682
**Femur‐LT**	Maximum dose (%)	0.0000	0.0144	0.1429	0.1583	0.3177	0.0421	0.0631	0.2094	0.1201
	Mean dose (%)	0.0000	0.0022	0.0101	0.5798	0.7615	0.9542	0.1118	0.9400	0.3883
	Minimum dose (%)	0.1249	0.2865	0.5871	0.8242	0.8768	0.9340	0.4703	0.6609	0.6645
**Femur‐RT**	Maximum dose (%)	0.0000	0.4516	0.6915	0.0677	0.0043	0.0010	0.1863	0.0716	0.0856
	Mean dose [%)	0.0000	0.0000	0.0001	0.0035	0.1629	0.0711	0.0036	0.0403	0.0236
	Minimum dose (%)	0.1061	0.5409	1.0000	0.7075	0.4057	0.5919	0.8840	0.4950	0.8847
**The percentage of statistically significant metrics at 0.01 *< p* ≤ 0.05**		**78%**	**44%**	**44%**	**33%**	**39%**	**44%**	**56%**	**50%**	**61%**
**The percentage of statistically significant metrics at *p* ≤ 0.01**		**78%**	**22%**	**44%**	**28%**	**33%**	**39%**	**44%**	**33%**	**50%**

Abbreviations: ART, adaptive radiation therapy; CTV, clinical target volume; PTV, planning target volume.

Table 3 shows that the majority of the results were statistically significant for the *non‐ART* treatment. Among ART plans the lowest *p*‐value was observed for *Cont.+DF* and offline adaptations, while the highest were observed for the remaining plan modification strategies. The results for PTV and CTV indicate a similar level of statistical significance. When considering organs at risk, the results for the rectum demonstrated a statistical significance similar to that of the bladder with the exception of minimum dose for which results corresponding to bladder were more significant compared to the rectum.

### 3.5 Time efficiency

The time required to manually complete key steps in the ART loop in the clinic was: (i) plan optimization ∼4 min 30 s.; (ii) dose calculation ∼1 min 30 s.; (iii) DIR ∼1 min. 50 s, for a total time of around 7 min 50 s per online plan adaptation for a single fraction. The limitation of our time estimation is that it did not include CBCT image acquisition, data processing, and possible verification step that may be required for a newly adapted treatment plan.

### 3.6 Qualitative assessment of image registration

Thorough visual inspection of image registration results did not reveal any major, non‐physical image deformations that could negatively impact the dosimetric results of ART strategies explored in this study. The alignment of the soft and bone tissues as well as external body contours that were inspected with overlays, checkerboards filter and dynamic magnifying window confirmed a high quality of image registrations. The deformation vector field was smooth without folding distortions, indicating that only realistic deformations of the patient anatomy occurred during the registration process.

## 4 DISCUSSION

*Offline* and *Offline*+*Online* plan adaptations resulted in the highest delivered dose to CTV and PTV compared to other ART strategies which was demonstrated by the majority of dose‐volume metrics (Figure 2). As explained in the Material and Methods section, *Offline* adaptation relies on the patient's anatomy from the previous fraction to modify the treatment plan that will be delivered in the next fraction. The possible issue in that approach is that if the magnitude of interfractional changes in the patient's anatomy is significant, the offline adapted treatment plan may not be able to correct the *non‐ART* plan as intended. However, literature findings have shown that at least in some situations, online and offline ART approaches can deliver similar dosimetric performance (which can be patient specific). For example, Qin et al. investigated 22 prostate cancer patients who underwent IMRT treatment (total dose of 64 Gy in 20 fractions) and found that both the online and offline adaptations performed similarly.^26^

Figure 2 also demonstrates that relative to CTV the dose coverage of the PTV is more sensitive to anatomical changes. The D_98%_ and D_99%_ for that structure were on average 5% and 6% lower for the unmodified plan. The importance of proper PTV coverage highlights the significance of ART application, as it has proven its ability to improve and maintain PTV coverage. Overall, both the dose and dose‐volume metrics shown in Figures 1 and 2 show that *non‐ART* plans delivered consistently lower doses to CTV and PTV compared to the *Planned* and all the ART plans.

Regarding the HI index (Figure 3), although we found that for CTV the daily plan re‐optimizations were generating satisfactory dose distributions, their combination in the dose accumulation step resulted in an even higher level of homogeneity for the majority of patients. Despite the promising results in the HI index for CTV, the accumulated dose distribution needs to be carefully examined in clinical practice. This is because the dose accumulation can result in the appearance of hot and cold spots depending on the spatial relation between daily dose distributions. This effect is most likely responsible for the presence of relatively high doses delivered to the bladder by the *Cont.+DF* adaptation as seen in Figure 4a (D_1%_, D_1cc_, and D_2%_) and Figure 4b (V_80%_ and V_95%_). It is also worth mentioning that for *Online* adaptations, a clinically acceptable value of HI was not associated with an increased dose in OARs. This is contrary to the findings of Banaei et al. who conducted a study based on 15 prostate cancer patients that were delivered IMRT treatment.^27^ Researchers reported inverse exponential relationships between the OAR sparing and HI, which might be the case due to the differences in dose delivery techniques. Banaei et al. used nine static IMRT beams, while this study utilized VMAT techniques with two arcs. Chow et al. showed that in the case of prostate cancer, VMAT compared to IMRT provides more desirable PTV coverage in terms of both HI (0.09 vs. 0.12) and CI index (0.94 vs. 0.89).^28^ It should also be noted that based on the data presented in the Figure 4, the *non‐ART* plans delivered lower radiation doses to both bladder and rectum compared to all other delivery approaches. Although it can be seen as a desirable outcome, the *non‐ART* plans are not optimal because as mentioned in the previous paragraph, both CTV and PTV coverage significantly differ from the planned dose.

It is also important to notice that as shown in Figure 5, the dose delivered by adapted plans to the right femoral head was noticeably lower than for the left femoral head. It is not very clear why the dose sparing is not approximately symmetrical for these two structures. The authors suspect that because the dose delivered to femoral heads was significantly lower than planning objective threshold, the observation of asymmetry is a form of systematic noise and an indication of limited priority given to those structures by the optimizer during plan re‐optimization. The direction of the first arc may also contribute to this, as the second arc (in the opposite direction) typically has a lesser dose impact than the first arc (i.e., the second arc “fine tunes” the dose). In the early adoption of VMAT in clinical practice, Hardcastle et al.^29^ reported such asymmetry but admitted that the reason was also not clear, further adding that gantry rotation direction did not affect the asymmetry of the dose distribution. Also, Tran et al.^23^ indicated that the left femoral head received a higher dose than the right femoral head however due to the fact that dose‐volume objectives were met, this observation was not discussed.

Figure 6a clearly shows the advantage of ART application in that the passing rate for D_99%_ in CTV was around 100% for the *Online*, *Online 1‐3‐5, and Online+DF* adaptations compared to 0% for the unaltered *non‐ART* plan. The 100% passing rate for the bladder for all plans, as shown in Figure 7a was anticipated because VMAT plans have been proven to be able to decrease the dose delivered to that organ very effectively (large ΔD∼60% on Figure 7b) in prostate cancer radiotherapy.^30^ Limiting the dose to the rectum by the application of various ART strategies was more challenging (small ΔD∼10% in Figure 7b, passing rate as low as 65% for D_20%_ in Figure 7a) due to the position, size, and the increased daily movement of the rectum. Also, the *Online* approach resulted in the highest passing rate for rectum planning objectives. The analysis of treatment planning objective passing rates and dose deviations ΔD shows that *Online* and *Online 1‐3‐5* strategies are very promising for ART adoption in the clinical environment. It can be noticed that the DF approaches resulted in the lowest absolute values of ΔD (Figure 6b) for target structures; however, compared to online strategies they are significantly more resource intensive thus may not be an optimal choice in practical ART applications.

It is also interesting to observe that the passing rate for PTV objective (D99% > 3800cGy, Figure 6a) was very low. In particular *non‐ART*, Onl*ine 1‐3‐5, DF 2–4, Offline+DF, Offline+Online, Offline* all had zero or close to zero passing rates. Figure 6b shows why this might be the case. We can see that even the original (*Planned*) plans barely meet that criteria (ΔD is almost equal to zero; 0.02%). This means that any, even the smallest random error related to any aspect of treatment delivery, patient positioning etc. could easily invalidate this particular objective. In our case, the source of this error could be very small random contouring variability. Only the most resource intensive strategies (*Cont.+DF*, *Online+DF*, and *Online*) were able to slightly mitigate this discrepancy.

One of the study limitations is that the comprehensive quantitative evaluation of the DIR algorithm as well as the spatial relationship between the registration error and its impact on the accuracy of dose estimation was beyond the scope of this work. However, it is expected to be a relatively small effect compared to the impact of the various adaptation strategies.^31^

Table 3 shows that the statistical significance of the results is, in general, higher for

*Online 1‐3‐5* plans compared to *Online* ART. Although overall the *Online* adaptation delivered a dosimetric performance slightly closer to the initially intended plan, the *Online 1‐3‐5* strategy is 40% faster due to fewer adaptations required. The trade‐off between the time efficiency and the dosimetric results presented can be useful for both busy clinics and centers with larger time allocation per treatment plan.

As mentioned in the introduction, ART is an active area of research; however, the number of papers reporting the comprehensive evaluation of various adaptation scenarios is limited. Often authors study just a single or few approaches.^32^, ^33^, ^34^, ^35^, ^36^ Therefore, we believe that our comprehensive approach brings value in evaluating ART focused on the two full arcs hypofractionated VMAT treatments for prostate cancer patients considered in this study. The evaluation of a variety of ART strategies will help to easier identify an ART approach that is best suited for individual clinics.

## 5 CONCLUSIONS

The aim of this research was to investigate and quantify the dosimetric benefits resulting from the application of several different adaptive radiation therapy strategies for hypofractionated VMAT treatments for prostate cancer patients. The findings of our work quantify these improvements and indicate that performing daily online adaptations, every fraction or every second fraction improves the dosimetric outcomes of delivered radiotherapy treatment compared to the plan that was created solely based on the pre‐treatment planning CT scan and was then delivered without accounting for interfractional changes in the patient's anatomy. The strategy of adapting every second fraction achieves nearly the same dosimetric benefit to the patient but with significantly reduced resources used and may represent the most clinically attractive strategy examined here for significantly hypofractionated prostate cancer patients.

## CONFLICT OF INTEREST

The authors declare that there is no conflict of interest that could be perceived as prejudicing the impartiality of the research reported.

## AUTHOR CONTRIBUTIONS

*Manuscript writing, deformable image registration, adaptation of treatment plans as well as acquisition, processing, analysis, and interpretation of data*: Pawel Siciarz. *Revising and providing the feedback to the work for important intellectual content and manuscript editing*: Boyd McCurdy. *Strong clinical contribution in the form of manual segmentation of anatomical structures on imaging data sets*: Nikesh Hanumanthappa. *Critical software support and data interpretation*: Eric Van Uytven.

## 1

**TABLE A1:** Maximum, mean, and minimum radiation doses with corresponding standard deviations for the reference and adaptive radiation therapy (ART) strategies

				Adaptive radiation therapy strategies							
										Offline	
Structure	Metric	Planned	non‐ART	Cont.+DF	DF 2‐4	Online+DF	Online	Online1‐3‐5	Offline+DF	+Online	Offline
**CTV**	Maximum dose (%)	105.28 ± 0.71	99.81 ± 0.25	105.69 ± 2.31	106.10 ± 2.07	105.98 ± 2.53	105.84 ± 2.35	105.81 ± 1.65	106.89 ± 2.40	107.83 ± 4.42	108.76 ± 3.64
	Mean dose (%)	102.12 ± 0.50	97.48 ± 0.31	103.32 ± 1.96	103.80 ± 1.88	103.49 ± 1.92	103.84 ± 2.37	103.93 ± 1.65	104.43 ± 2.10	105.65 ± 4.36	106.71 ± 3.39
	Minimum dose (%)	99.32 ± 0.59	94.47 ± 1.56	100.89 ± 2.30	101.49 ± 1.51	101.49 ± 1.94	101.68 ± 1.96	101.87 ± 1.34	100.96 ± 2.39	101.71 ± 2.09	101.10 ± 2.20
**PTV**	Maximum dose (%)	105.91 ± 0.64	100.00 ± 0.00	106.67 ± 2.32	106.77 ± 20	106.82 ± 2.47	106.07 ± 2.38	105.95 ± 1.60	107.59 ± 2.29	108.10 ± 4.38	108.89 ± 3.60
	Mean dose (%)	101.37 ± 0.38	96.17 ± 0.58	102.26 ± 1.44	102.51 ± 1.28	102.39 ± 1.43	102.54 ± 1.76	102.60 ± 1.19	103.07 ± 1.60	103.98 ± 3.41	104.72 ± 2.63
	Minimum dose (%)	90.15 ± 0.70	74.77 ± 8.81	86.17 ± 6.97	85.99 ± 4.71	85.01 ± 9.18	84.73 ± 10.14	84.88 ± 6.03	84.51 ± 7.19	81.86 ± 10.06	82.92 ± 6.65
**Bladder**	Maximum dose (%)	105.44 ± 0.78	99.17 ± 0.61	106.25 ± 2.39	105.94 ± 1.85	106.08 ± 2.41	105.36 ± 2.38	105.27 ± 1.53	106.78 ± 2.54	106.93 ± 4.13	107.67 ± 3.20
	Mean dose (%)	15.67 ± 6.52	14.24 ± 5.63	16.59 ± 7.86	15.68 ± 6.90	15.82 ± 7.38	15.63 ± 7.09	15.49 ± 6.47	16.06 ± 7.08	15.81 ± 6.87	15.74 ± 6.59
	Minimum dose (%)	1.32 ± 1.06	0.65 ± 1.23	0.75 ± 1.63	0.74 ± 1.48	0.74 ± 1.54	0.74 ± 1.48	0.72 ± 1.42	0.75 ± 1.51	0.73 ± 1.44	0.73 ± 1.43
**Rectum**	Maximum dose (%)	103.79 ± 1.03	98.36 ± 1.02	104.86 ± 2.29	104.28 ± 1.22	103.97 ± 1.84	104.29 ± 2.17	104.29 ± 1.22	104.75 ± 1.98	105.78 ± 4.31	106.51 ± 3.58
	Mean dose (%)	38.60 ± 7.67	38.89 ± 7.77	38.86 ± 7.63	39.69 ± 7.21	39.10 ± 7.90	39.54 ± 7.77	40.26 ± 7.71	39.49 ± 7.72	40.20 ± 8.08	41.06 ± 8.18
	Minimum dose (%)	2.51 ± 0.98	2.20± 1.14	2.37 ± 1.30	2.36 ± 1.21	2.37 ± 1.20	2.35 ± 1.19	2.35 ± 1.20	2.40 ± 1.22	2.38 ± 1.23	2.42 ± 1.24
**Femur‐LT**	Maximum dose (%)	33.45 ± 5.24	31.23 ± 4.97	35.57 ± 5.65	34.26 ± 5.39	34.35 ± 5.28	34.11 ± 5.06	32.44 ± 4.58	34.80 ± 5.50	34.43 ± 5.16	34.41 ± 5.37
	Mean dose (%)	13.74 ± 2.46	13.16 ± 2.23	15.16 ± 2.76	14.55 ± 2.54	13.96 ± 2.52	13.62 ± 2.43	13.76 ± 2.25	14.40 ± 2.57	13.77 ± 2.39	14.03 ± 2.41
	Minimum dose (%)	1.10 ± 0.34	1.01 ± 0.43	1.17 ± 0.53	1.13 ± 0.50	1.11 ± 0.53	1.11 ± 0.52	1.09 ± 0.49	1.15 ± 0.53	1.13 ± 0.53	1.13 ± 0.52
**Femur‐RT**	Maximum dose (%)	35.36 ± 6.75	33.48 ± 6.26	35.86 ± 5.54	35.17 ± 5.45	33.88 ± 4.91	32.66 ± 4.64	33.06 ± 4.92	34.26 ± 5.56	33.74 ± 4.59	33.80 ± 5.36
	Mean dose (%)	13.65 ± 2.23	12.96 ± 2.07	15.51 ± 2.48	14.54 ± 2.28	14.67 ± 2.53	14.09 ± 2.47	14.04 ± 2.30	14.67 ± 2.54	14.36 ± 2.51	14.43 ± 2.64
	Minimum dose (%)	1.11 ± 0.36	1.01 ± 0.46	1.16 ± 0.60	1.11 ± 0.52	1.09 ± 0.53	1.06 ± 0.52	1.08 ± 0.51	1.12 ± 0.54	1.07 ± 0.52	1.10 ± 0.53

Abbreviations: CTV, clinical target volume; DF, dose feedback; PTV, planning target volume.

**TABLE A2:** Dose and dose‐volume metrics with corresponding standard deviations for the reference and adaptive radiation therapy (ART) strategies

			Adaptive radiation therapy strategies								
Structure	Metric	Planned	non‐ART	Cont.+DF	DF 2‐4	Online+DF	Online	Online 1‐3‐5	Offline+DF	Offline+Online	Offline
**CTV**	D1% (cGy)	4149 ± 23	3953 ± 10	4184 ± 83	4201 ± 83	4188 ± 83	4195 ± 96	4198 ± 67	4230 ± 90	4274 ± 180	4314 ± 144
	D2% (cGy)	4142 ± 23	3946 ± 10	4178 ± 83	4195 ± 83	4181 ± 81	4189 ± 97	4192 ± 67	4222 ± 91	4268 ± 180	4309 ± 143
	D5% (cGy)	4131 ± 22	3937 ± 11	4169 ± 82	4185 ± 83	4171 ± 80	4181 ± 96	4184 ± 67	4212 ± 91	4259 ± 179	4301 ± 143
	D50% (cGy)	4084 ± 20	3898 ± 13	4132 ± 78	4152 ± 78	4139 ± 77	4153 ± 94	4156 ± 66	4180 ± 90	4227 ± 177	4273 ± 139
	D95% (cGy)	4042 ± 21	3864 ± 16	4099 ± 77	4119 ± 62	4112 ± 75	4125 ± 94	4131 ± 65	4132 ± 77	4185 ± 167	4220 ± 133
	D98% (cGy)	4033 ± 21	3854 ± 18	4090 ± 79	4110 ± 60	4105 ± 76	4116 ± 95	4123 ± 65	4117 ± 80	4164 ± 147	4192 ± 117
	D99% (cGy)	4027 ± 21	3846 ± 23	4083 ± 81	4105 ± 60	4099 ± 76	4110 ± 95	4118 ± 65	4106 ± 83	4146 ± 124	4166 ± 94
	V100%	99.87 ± 0.17	0.00 ± 0.00	99.87 ± 0.36	99.99 ± 0.03	99.99 ± 0.03	99.97 ± 0.10	100 ± 0.00	99.78 ± 0.75	99.93 ± 0.33	99.88 ± 0.36
	V105%	0.15 ± 0.49	0.00 ± 0.00	11.37 ± 30.55	18.60 ± 36.63	15.19 ± 33.66	15.52 ± 35.04	14.88 ± 32.66	28.99 ± 44.35	40.15 ± 46	61.29 ± 42.73
	HI [%]	0.03 ± 0.00	0.02 ± 0.00	0.02 ± 0.01	0.02 ± 0.01	0.02 ± 0.00	0.02 ± 0.00	0.02 ± 0.00	0.02 ± 0.02	0.02 ± 0.01	0.03 ± 0.02
**PTV**	D1% (cGy)	4158 ± 21	3954 ± 8	4198 ± 81	4207 ± 80	4198 ± 80	4197 ± 95	4200 ± 66	4238 ± 88	4275 ± 179	4313 ± 143
	D2% (cGy)	4149 ± 20	3947 ± 9	4188 ± 79	4199 ± 80	4188 ± 79	4191 ± 96	4193 ± 66	4229 ± 88	4268 ± 179	4307 ± 143
	D5% (cGy)	4135 ± 19	3936 ± 10	4175 ± 79	4187 ± 80	4175 ± 78	4181 ± 95	4184 ± 66	4215 ± 89	4258 ± 178	4298 ± 142
	D50% (cGy)	4075 ± 17	3887 ± 13	4123 ± 74	4138 ± 64	4131 ± 75	4143 ± 92	4146 ± 63	4168 ± 83	4212 ± 169	4254 ± 129
	D95% (cGy)	3880 ± 8	3591 ± 110	3880 ± 8	3880 ± 8	3880 ± 8	3880 ± 8	3880 ± 8	3880 ± 8	3880 ± 8	3880 ± 8
	D98% (cGy)	3828 ± 3	3459 ± 162	3799 ± 60	3800 ± 37	3788 ± 98	3786 ± 115	3788 ± 63	3783 ± 68	3742 ± 148	3745 ± 119
	D99% (cGy)	3801 ± 1	3372 ± 203	3740 ± 116	3743 ± 71	3722 ± 179	3717 ± 206	3723 ± 115	3714 ± 127	3652 ± 228	3659 ± 179
	V100%	83.02 ± 2.33	0.00 ± 0.00	85.12 ± 2.60	85.36 ± 2.12	85.57 ± 2.80	85.13 ± 3.03	85.89 ± 1.93	86.09 ± 2.58	87.25 ± 3.06	87.64 ± 3.1
	V105%	0.14 ± 0.30	0.00 ± 0.00	9.11 ± 22.70	12.72 ± 24.46	11.77 ± 25.09	11.83 ± 26.59	10.56 ± 22.87	21.79 ± 32.42	30.14 ± 35.16	44.86 ± 32.99
	V95%	99.02 ± 0.04	81.99 ± 4.62	98.32 ± 0.80	98.14 ± 0.70	98.22 ± 0.83	98.36 ± 0.89	98 ± 0.67	97.92 ± 0.71	97.67 ± 0.98	97.60 ± 10
	CI [%]	0.96 ± 0.01	0.86 ± 0.21	0.93 ± 0.03	0.92 ± 0.04	0.94 ± 0.03	0.94 ± 0.03	0.93 ± 0.03	0.93 ± 0.08	0.91 ± 0.06	0.90 ± 0.05
	HI [%]	0.08 ± 0.00	0.13 ± 0.04	0.09 ± 0.03	0.10 ± 0.02	0.10 ± 0.04	0.10 ± 0.04	0.10 ± 0.03	0.11 ± 0.03	0.12 ± 0.07	0.13 ± 0.05
**Bladder**	D1% (cGy)	3925 ± 226	3598 ± 290	3938 ± 235	3893 ± 243	3932 ± 233	3914 ± 244	3883 ± 256	3959 ± 192	3927 ± 254	3911 ± 196
	D1cc (cGy)	4099 ± 40	3832 ± 108	4135 ± 71	4115 ± 75	4135 ± 72	4126 ± 73	4107 ± 80	4162 ± 92	4164 ± 109	4181 ± 109
	D2% (cGy)	3577 ± 405	3266 ± 457	3616 ± 423	3540 ± 411	3580 ± 433	3563 ± 439	3528 ± 425	3611 ± 390	3587 ± 440	3538 ± 371
	V15%	25.73 ± 13.55	24.12 ± 11.59	28.55 ± 16.38	26.34 ± 14.42	26.53 ± 15.55	26.09 ± 14.86	25.98 ± 13.77	26.94 ± 15.10	26.23 ± 14.30	26.39 ± 13.72
	V20%	22.61 ± 12.05	20.86 ± 10.15	24.56 ± 14.69	22.72 ± 12.76	22.93 ± 13.92	22.55 ± 13.3	22.41 ± 12.12	23.26 ± 13.42	22.72 ± 12.86	22.78 ± 12.23
	V50%	10.12 ± 5.09	8.79 ± 4.25	10.63 ± 6.33	10.02 ± 5.44	10.13 ± 5.84	10.02 ± 5.53	9.89 ± 4.91	10.34 ± 5.51	10.22 ± 5.40	10.12 ± 5.13
	V80%	3.60 ± 1.84	2.75 ± 1.56	4.07 ± 2.68	3.61 ± 2.03	3.78 ± 2.31	3.67 ± 2.14	3.50 ± 1.80	3.87 ± 2.15	3.75 ± 2.07	3.6 ± 1.91
	V95%	1.86 ± 1.02	0.79 ± 0.70	2.11 ± 1.66	1.74 ± 1.10	1.99 ± 1.36	1.88 ± 1.18	1.68 ± 0.96	1.99 ± 1.20	1.90 ± 1.16	1.68 ± 1.00
**Rectum**	D1% (cGy)	3979 ± 71	3818 ± 101	4014 ± 128	4010 ± 69	3999 ± 96	4020 ± 111	4038 ± 75	3996 ± 102	4065 ± 181	4084 ± 138
	D1cc (cGy)	3942 ± 82	3798 ± 121	3973 ± 142	3975 ± 79	3958 ± 104	3978 ± 111	4003 ± 73	3953 ± 115	4025 ± 178	4047 ± 146
	D2% (cGy)	3883 ± 114	3744 ± 149	3903 ± 177	3914 ± 108	3888 ± 133	3914 ± 145	3944 ± 115	3889 ± 130	3953 ± 199	3976 ± 143
	V15%	66.68 ± 15.64	67.8 ± 15.55	70.02 ± 14.49	68.92 ± 14.64	68.99 ± 14.49	68.75 ± 14.44	68.75 ± 14.76	69.11 ± 14.64	68.94 ± 14.28	69.10 ± 14.58
	V20%	64.22 ± 15.44	65.15 ± 15.43	67.01 ± 14.33	66.15 ± 14.51	66.22 ± 14.37	65.96 ± 14.27	65.96 ± 14.62	66.34 ± 14.52	66.09 ± 14.12	66.27 ± 14.49
	V50%	38.29 ± 9.37	37.41 ± 9.96	35.67 ± 9.69	37.91 ± 9.07	37.91 ± 10.55	39.28 ± 10.48	40.39 ± 10.22	38.11 ± 9.83	40.54 ± 11.22	42.04 ± 10.92
	V80%	8.84 ± 2.45	10.04 ± 4.49	8.91 ± 2.65	10.25 ± 3.13	8.79 ± 2.60	9.09 ± 2.53	10.37 ± 3.10	9.36 ± 2.73	9.80 ± 2.96	11.05 ± 2.93
	V95%	3.19 ± 1.36	2.43 ± 2.01	3.32 ± 1.51	3.67 ± 1.50	3.12 ± 1.19	3.41 ± 1.40	3.94 ± 1.68	3.13 ± 1.26	3.76 ± 1.91	4.04 ± 1.67
**Femur‐LT**	D1% (cGy)	1153 ± 164	1090 ± 152	1270 ± 187	1209 ± 176	1211 ± 169	1198 ± 161	1158 ± 145	1232 ± 182	1210 ± 161	1211 ± 168
	D1cc (cGy)	1190 ± 174	1123 ± 162	1305 ± 191	1243 ± 182	1247 ± 176	1236 ± 167	1190 ± 151	1268 ± 186	1248 ± 169	1247 ± 176
	D2% (cGy)	1100 ± 155	1042 ± 142	1216 ± 177	1157 ± 164	1156 ± 160	1142 ± 151	1108 ± 135	1179 ± 172	1154 ± 150	1157 ± 157
	D5% (cGy)	1009 ± 140	957 ± 125	1118 ± 162	1063 ± 146	1058 ± 146	1041 ± 139	1017 ± 120	1082 ± 156	1051 ± 134	1058 ± 140
**Femur‐RT**	D1% (cGy)	1214 ± 219	1155 ± 207	1286 ± 189	1237 ± 183	1203 ± 171	1157 ± 165	1172 ± 166	1216 ± 191	1190 ± 160	1194 ± 186
	D1cc (cGy)	1255 ± 226	1194 ± 213	1320 ± 194	1272 ± 186	1236 ± 175	1191 ± 169	1204 ± 170	1249 ± 195	1224 ± 165	1230 ± 190
	D2% (cGy)	1154 ± 204	1099 ± 192	1235 ± 179	1184 ± 173	1154 ± 162	1108 ± 157	1124 ± 158	1165 ± 181	1139 ± 153	1143 ± 178
	D5% (cGy)	1049 ± 172	998 ± 161	1144 ± 163	1090 ± 158	1067 ± 148	1021 ± 145	1036 ± 145	1075 ± 164	1048 ± 141	1052 ± 162

Abbreviation: DF, dose feedback.

**TABLE A3:** The results of paired, two‐tailed, *t*‐test for D_max_, D_min_, and D_mean_. The fields for which 0.01 < *p* ≤ 0.05 were highlighted in green (i.e., significant), while those with *p* ≤ 0.01 were highlighted in red (i.e., very significant)

			Adaptive radiation therapy strategies							
			Cont.		Online		Online	Offline	Offline	
Structure	Metric	non‐ART	+DF	DF 2–4	+DF	Online	1‐3‐5	+DF	+Online	Offline
**CTV**	D1% (cGy)	0.0000	0.0763	0.0197	0.0535	0.0550	0.0078	0.0011	0.0075	0.0001
	D2% (cGy)	0.0000	0.0654	0.0180	0.0488	0.0459	0.0057	0.0011	0.0068	0.0001
	D5% (cGy)	0.0000	0.0514	0.0146	0.0398	0.0349	0.0035	0.0011	0.0057	0.0001
	D50% (cGy)	0.0000	0.0146	0.0021	0.0061	0.0050	0.0002	0.0002	0.0022	0.0000
	D95% (cGy)	0.0000	0.0043	0.0001	0.0007	0.0011	0.0000	0.0001	0.0014	0.0000
	D98% (cGy)	0.0000	0.0047	0.0000	0.0006	0.0011	0.0000	0.0002	0.0009	0.0000
	D99% (cGy)	0.0000	0.0065	0.0000	0.0006	0.0011	0.0000	0.0004	0.0005	0.0000
	V100%	0.0000	0.9355	0.0047	0.0042	0.0332	0.0025	0.6075	0.4931	0.8273
	V105%	0.1734	0.1173	0.0367	0.0606	0.0650	0.0585	0.0090	0.0010	0.0000
	HI [%]	0.0001	0.0041	0.0071	0.0000	0.0000	0.0000	0.6951	0.5128	0.9334
										
**PTV**	D1% (cGy)	0.0000	0.0451	0.0204	0.0441	0.0932	0.0179	0.0010	0.0111	0.0002
	D2% (cGy)	0.0000	0.0411	0.0179	0.0422	0.0698	0.0108	0.0009	0.0094	0.0001
	D5% (cGy)	0.0000	0.0360	0.0140	0.0337	0.0456	0.0051	0.0009	0.0073	0.0001
	D50% (cGy)	0.0000	0.0092	0.0007	0.0039	0.0043	0.0001	0.0001	0.0021	0.0000
	D95% (cGy)	0.0000	0.2591	0.3535	0.1183	0.9169	0.2698	0.5916	0.0912	0.1224
	D98% (cGy)	0.0000	0.0380	0.0017	0.0749	0.1145	0.0091	0.0065	0.0161	0.0049
	D99% (cGy)	0.0000	0.0316	0.0018	0.0635	0.0840	0.0072	0.0065	0.0089	0.0022
	V100%	0.0000	0.0056	0.0067	0.0024	0.0119	0.0007	0.0015	0.0001	0.0000
	V105%	0.0474	0.0935	0.0334	0.0524	0.0644	0.0563	0.0076	0.0012	0.0000
	V95%	0.0000	0.0010	0.0000	0.0004	0.0035	0.0000	0.0000	0.0000	0.0000
	CI [%]	0.0606	0.0048	0.0018	0.0100	0.1022	0.0001	0.2266	0.0015	0.0003
	HI [%]	0.0001	0.0340	0.0079	0.0520	0.0896	0.0088	0.0011	0.0081	0.0004
										
**Bladder**	D1% (cGy)	0.0000	0.4900	0.0239	0.6020	0.3387	0.0106	0.0914	0.9240	0.4712
	D1cc (cGy)	0.0000	0.0110	0.2207	0.0240	0.1030	0.5861	0.0061	0.0136	0.0025
	D2% (cGy)	0.0001	0.2156	0.1071	0.9276	0.6059	0.0393	0.2351	0.7476	0.0942
	V15%	0.0296	0.0311	0.4975	0.4878	0.7160	0.7063	0.2763	0.5604	0.4115
	V20%	0.0159	0.1097	0.8936	0.7677	0.9521	0.7493	0.5163	0.8967	0.8167
	V50%	0.0017	0.3624	0.7531	0.9740	0.7937	0.2712	0.5346	0.7781	0.9954
	V80%	0.0015	0.1048	0.9235	0.3488	0.6072	0.2370	0.1117	0.2749	0.9960
	V95%	0.0000	0.2292	0.0769	0.3171	0.8027	0.0079	0.2408	0.5762	0.0184
										
**Rectum**	D1% (cGy)	0.0000	0.1138	0.0258	0.2372	0.0527	0.0000	0.3613	0.0316	0.0029
	D1cc (cGy)	0.0000	0.1959	0.0328	0.3520	0.0683	0.0000	0.5468	0.0326	0.0020
	D2% (cGy)	0.0002	0.4637	0.0688	0.8081	0.1469	0.0000	0.7848	0.0680	0.0045
	V15%	0.0126	0.0001	0.0002	0.0009	0.0016	0.0002	0.0001	0.0012	0.0001
	V20%	0.0266	0.0002	0.0002	0.0018	0.0047	0.0008	0.0001	0.0052	0.0002
	V50%	0.2669	0.0065	0.5856	0.6954	0.3971	0.0352	0.8517	0.1745	0.0041
	V80%	0.1341	0.7808	0.0097	0.8991	0.3480	0.0005	0.1393	0.0518	0.0000
	V95%	0.0518	0.5697	0.0972	0.7096	0.1924	0.0001	0.7728	0.0747	0.0093
**Femur‐LT**	D1% (cGy)	0.0000	0.0004	0.0049	0.0214	0.0643	0.7559	0.0032	0.0442	0.0121
	D1cc (cGy)	0.0000	0.0006	0.0079	0.0245	0.0665	0.9993	0.0038	0.0526	0.0146
	D2% (cGy)	0.0000	0.0004	0.0041	0.0229	0.0817	0.6301	0.0030	0.0485	0.0111
	D5% (cGy)	0.0000	0.0006	0.0058	0.0395	0.1770	0.5870	0.0045	0.0949	0.0169
**Femur‐RT**	D1% (cGy)	0.0000	0.0067	0.1997	0.6714	0.0439	0.0461	0.9645	0.3893	0.4811
	D1cc (cGy)	0.0000	0.0155	0.3434	0.5038	0.0311	0.0240	0.8257	0.3048	0.3913
	D2% (cGy)	0.0000	0.0018	0.0741	0.9893	0.0763	0.1097	0.6797	0.5635	0.6704
	D5% (cGy)	0.0000	0.0001	0.0064	0.4183	0.1949	0.3828	0.2425	0.9733	0.8759
**The percentage of statistically significant metrics at 0.01 < *p*≤ 0.05**		**88%**	**63%**	**66%**	**44%**	**29%**	**66%**	**49%**	**51%**	**68%**
**The percentage of statistically significant metrics at *p* ≤ 0.01**		**76%**	**41%**	**46%**	**24%**	**17%**	**49%**	**49%**	**34%**	**56%**

Abbreviations: ART, adaptive radiation therapy; CTV, clinical target volume; PTV, planning target volume.
